# Livedo racemosa and spontaneous retinal detachment as presenting signs of paraneoplastic sarcoid vasculitis

**DOI:** 10.1016/j.jdcr.2025.08.009

**Published:** 2025-08-19

**Authors:** Nadean F. Alnajjar, Olivia M.T. Davies, Ashley Gochoco, Stephen R. Lyle, Christopher Iriarte

**Affiliations:** a Harvard Medical School, Boston, Massachusetts; b Department of Dermatology, Beth Israel Deaconess Medical Center, Boston, Massachusetts; c Harvard Combined Residency Program, Boston, Massachusetts; d Department of Pathology, Beth Israel Deaconess Medical Center, Boston, Massachusetts; e Department of Dermatology, Harvard Medical School, Boston, Massachusetts

**Keywords:** malignancy, paraneoplastic sarcoidosis

## Introduction

Sarcoidosis is a multisystem inflammatory disease characterized by noncaseating granulomatous inflammation. Numerous associations have been documented in the literature, including sarcoidosis occurring as a paraneoplastic phenomenon.^1^ Herein, we describe a unique cutaneous and ocular presentation of sarcoid vasculitis that was ultimately secondary to myelodysplastic syndrome (MDS) that transformed into acute myeloid leukemia (AML). This case underscores the importance of maintaining a high clinical suspicion for sarcoidosis in patients presenting with pathology across multiple organ systems as well as considering malignancy as an underlying driver.

## Case

A 78-year-old female presented with 5 weeks of progressive vision loss as well as bilateral lower extremity edema with an associated rash. She had a past medical history of hypertension and a strong family history of malignancy. She did not endorse any other systemic symptoms.

**Fig 1:**
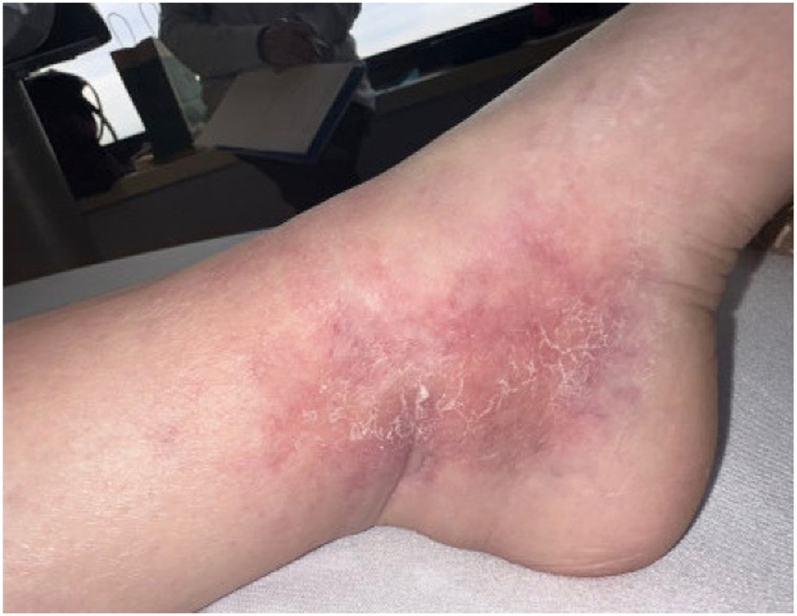
Livedo racemosa over the ankles.

**Fig 2:**
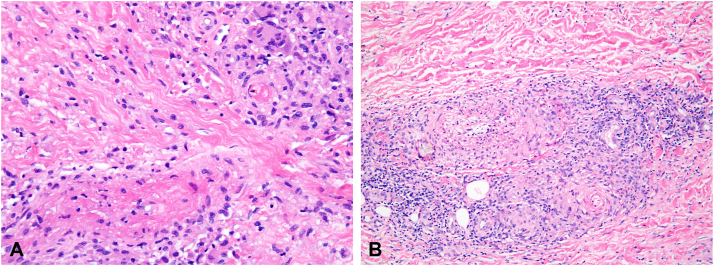
Pandermal acute necrotizing vasculitis. **A,** Necrotic and thrombosed vessel with surrounding lymphohistiocytic inflammation and multinucleated giant cells in the superficial dermis (hematoxylin-eosin, original magnification ×40). **B,** Necrotic vessel in the deep dermis with surrounding granulomatous and lymphohistocytic inflammation with eosinophils (hematoxylin-eosin, original magnification ×20).

An ophthalmology exam revealed bilateral serous retinal detachment, choroidal enhancement, and optic perineuritis. The skin exam was notable for 2+ pitting edema and asymmetric reticular violaceous patches consistent with livedo racemosa over the bilateral ankles (Fig 1). Histopathology from a skin punch biopsy of the left ankle revealed a necrotizing vasculitis with surrounding granulomatous inflammation, thrombosis, and multinucleated giant cells extending from the superficial to deep dermis (Fig 2).

Positron emission tomography/computed tomography demonstrated avidity in the bilateral lower extremity neurovascular bundles consistent with vasculitis as well as numerous lesions in the liver. Liver biopsy demonstrated non-necrotizing histiocytic granulomas without evidence of infection or malignancy. Further work-up was notable for an elevated serum angiotensin-converting enzyme level (99.9 U/L; reference: 9-67 U/L), positive antinuclear antibody (titer 1:640; speckled pattern), negative antineutrophil cytoplasmic antibody panel, leukopenia, and anemia. Her constellation of symptoms, including bicytopenia and unusual ocular findings, raised the question of an unidentified primary neoplasm, and she underwent bone marrow biopsy with subsequent genetic testing. These studies revealed 10% to 15% myeloblasts along with *ETV6* and *TP53* deletions, ultimately rendering a diagnosis of MDS.

Treatment was initiated with intravenous pulse-dose methylprednisolone and steroidal eye drops, followed by a prolonged oral prednisone taper, with resolution of rash and 50% recovery of vision. She was also started on azacitidine for her MDS. Unfortunately, her MDS eventually evolved to AML, prompting a switch in therapy.

## Discussion

This case emphasizes the importance of maintaining a high level of clinical suspicion for atypical presentations of sarcoidosis and underscores the critical role dermatologists play as diagnosticians on a multidisciplinary team.

Paraneoplastic sarcoidosis refers to sarcoidosis typically diagnosed within 1 year of a malignancy.^1^ A single-center study of sarcoidosis cases over 6 years found that 17.3% were associated with a malignancy, with gastrointestinal carcinoma being the most common (20%), followed by hematologic malignancies (18%).^2^

Cutaneous manifestations of sarcoidosis may be more commonly observed in paraneoplastic cases (over 50%) than they are in nonmalignancy-associated cases (20% to 25%), with skin involvement often the initial presenting sign.^3^ Sarcoid vasculitis can exhibit diverse dermatologic findings, including ulcerations, papules, erythematous nodules, infiltrative plaques, and livedoid changes.^3^^,^^4^ The finding of cutaneous granulomatous vasculitis as seen in our patient is a rare presentation, with an incidence of only 4.5% among all sarcoidosis cases.^4^

The occurrence of sarcoidosis over the age of 70 is rare and should signal that further investigation for an underlying malignancy is warranted.^3^ Our patient exhibited an exudative retinal detachment, which suggests an inflammatory, vascular, or neoplastic cause.^5^ The proposed mechanism involves disruption of the osmotic pump as regulated by the retinal pigment epithelium, resulting in exudative fluid buildup in the subretinal space.^5^ Ocular paraneoplastic sarcoidosis can impact any region of the eye and often presents bilaterally.^6^^,^^7^ In this case, the bilaterality and patient’s positive response to corticosteroids (prior to any MDS-directed therapy) in the form of partial vision recovery support an underlying inflammatory driver of this process.

Corticosteroids remain the first-line treatment for sarcoidosis, although treatment of the underlying malignancy is critical in paraneoplastic cases.^8^ In 1 study, El-Khalawany et al reported complete resolution of cutaneous sarcoid symptoms in 7 of 12 patients when treated for the primary malignancy.^8^ In our patient, it is likely that both corticosteroids and MDS-directed azacitidine therapy contributed to the improvement of her paraneoplastic sarcoidosis. The patient remained clinically stable with evidence of hematopoietic recovery, including rising hemoglobin levels and resolution of leukopenia, and experienced no recurrence of vision loss or livedo racemosa. Her disease unfortunately later progressed to AML without documented recurrence of her paraneoplastic sarcoidal features; however, her oncologic regimen was adjusted at this time.

Sarcoidosis remains a diagnostic challenge given its heterogenous clinical presentation. Our observation adds a unique presentation of paraneoplastic sarcoid vasculitis and emphasizes the importance of working up associated malignancy.

## Conflicts of interest

None disclosed.
